# Analysis of clinical characteristics of 2243 with positive anti‐gastric parietal cell antibody

**DOI:** 10.1002/jcla.23264

**Published:** 2020-02-28

**Authors:** Yaping Guo, Yingxia Hao, Xiaoyan Li, Xin Liu, Ying Liang, Wenjie Song, Shuqin Guo

**Affiliations:** ^1^ Department of Clinical Laboratory Baoding NO.1 Central Hospital Baoding China; ^2^ Department of Gastroenterology Baoding NO.1 Central Hospital Baoding China; ^3^ Chengde Medical University Chengde China; ^4^ Department of Endocrinology Baoding NO.1 Central Hospital Baoding China

**Keywords:** autoimmune gastritis, clinical characteristics, parietal cell antibody, predictive value

## Abstract

**Background:**

To facilitate the early detection of chronic diseases, we analyzed the clinical characteristics of anti‐gastric parietal cell antibody (PCA)‐positive population, revealed the early characteristics of the population.

**Methods:**

According to the retrospective analysis, current situation investigation and comparative analysis of the clinical characteristics and medical history of the subjects, the comparison between the groups was performed.

**Result:**

(a) The positive rate of PCA detection in department of gastroenterology in our hospital was 35.80%. Among the individuals who underwent PCA, esophagogastroduodenoscopy (EGD) and pathological examination at the same time, 33.59% of the patients with PCA positive were diagnosed as atrophic gastritis by gastroscopy, which was much higher than 9.09% of the patients with PCA negative. (b) The incidence of gastroesophageal reflux, hypertension, ischemic heart disease (IHD) and cerebral ischemia in PCA‐positive population were 65.45%, 81.63%, 15.43%, and 31.61%, respectively, which were significantly higher than those in the control group. (c) The incidence rates of decreased red blood cells (RBC) and increased homocysteine (HCY) in laboratory‐related tests were 38.30% and 69.15%, respectively, which were much higher than those in control group.

**Conclusion:**

PCA has predictive value for a variety of chronic diseases and timely detection is of great significance.

## 1 INTRODUCTION

The prevalence of digestive system diseases is high, and the outpatient rate with mild symptoms in the early stage is low. Due to the lack of effective diagnosis and treatment, the rate of misdiagnosis and missed diagnosis is also high. It has been reported that the misdiagnosis rate of disease such as inflammatory bowel disease in China is 36.8% and the missed diagnosis rate is as high as 60.9%.^1^ The digestive system is not only the guarantee system of energy supply for human activities, but also the material guarantee system of metabolism of the body. Its damage is very easy to secondary to other systemic disorders.

Parietal cell antibody (PCA) is an important member of autoantibody family. PCA is an immunoglobulin secreted by plasma cells against H^+^/K^+^‐ATP enzyme and gastrin receptor on the surface of gastric parietal cells.^2^ Once the PCA antibody is produced, it reacts with its target antigen, causing progressive damage to gastric parietal cells and their functions. Finally, autoimmune atrophic gastritis (AAG) is formed, which affects its secretion of H + and internal factors, and the absorption of vitamin B_12_ and folic acid. Vitamin B_12_ is a methyltransferase cofactor and an important material basis for the methylation of many substances and the redox reaction in vivo. It is involved in the metabolism of many important substances. The decrease in gastric acid secretion prevents the reduction of Fe3^+^ to Fe2^+^ in food. Hemoglobin synthesis disorder leads to iron deficiency anemia. Lack of vitamin B_12_ and endocrine factors lead to obstruction of nucleic acid synthesis and cell mitosis, which eventually lead to pernicious anemia. When vitamin B_12_ is deficient, the methionine circulation is blocked, and HCY cannot be converted into methionine and excreted out of the body, which results in the increase of plasma HCY level and eventually leads to H‐type hypertension. Vitamin B_12_ deficiency can also affect the metabolism of choline, epinephrine, norepinephrine, and other non‐nutrients, and secondary to a variety of diseases. Therefore, PCA is the initiator of many chronic diseases and exists in the serum of patients before symptoms appear. Early detection is of great significance for the prevention and treatment of many chronic diseases.

## 2 MATERIALS AND METHODS

### 2.1 Subjects

PCA‐positive group: In the past 5 years, PCA was detected in 6269 patients in the department of gastroenterology, with an average age of 57 ± 14 (age range 29‐82), including 2780 males and 3489 females, male: female = 1:1.26. The standard for admission was the occurrence of one or more digestive system symptoms such as nausea, vomiting, anorexia, acid reflux, belching, abdominal pain, and abdominal distention and one or more digestive system diseases. Those who are positive for PCA are the positive group. The study was approved by the Ethics Committee of Baoding NO.1 central hospitals (IRB: [2019]011), and informed consent was acquired from each individual.

PCA‐negative group: According to the age and sex composition of the above group objects, 400 PCA‐negative patients were stratified sampling, with an average age was 56 ± 12 (age range 27‐81), including 180 males and 220 females, male: female = 1:1.22. The standard for admission was the occurrence of one or more digestive system symptoms such as nausea, vomiting, anorexia, acid reflux, belching, abdominal pain, and abdominal distention and one or more digestive system diseases.

Physical examination group: According to the age and sex composition of the above group objects, 400 cases of physical examination population were stratified sampling to detect PCA, with an average age was 55 ± 11 (age range 29‐80), including 195 males and 205 females, male:female = 1:1.05.

There was no significant difference in the baseline characteristics of age and sex among the three groups.

### 2.2 Methods

#### 2.2.1 Research methods

The medical records of patients with PCA results were collected and analyzed from January 2015 to June 2019, and the clinical characteristics and medical history of PCA‐positive patients were retrospectively analyzed and investigated, which compared with the control group.

#### 2.2.2 Detection methods

Parietal cell antibody was detected in venous serum by indirect immunofluorescence assay and operated strictly in accordance with the standard operating procedures. We took double–blind reading. The titer of PCA > 100 was positive.

#### 2.2.3 Diagnostic criteria

Diagnostic criteria of essential hypertension according to ”Guidelines for Prevention and treatment of Hypertension in China 2010”: Blood pressure was measured three times in different days, systolic blood pressure ≥18.62 kpa (140 mm Hg), and/or diastolic blood pressure ≥11.97 kpa (90 mm Hg).The criteria for the decrease in red blood cells: male: 4.0‐5.5 × 10^12^/L; female: 3.5‐5.0 × 10^12^/L. The criteria of Hcy increase is serum Hcy ≥ 15 umol/L in fasting state. Gastroesophageal reflux: Gastroesophageal reflux disease and esophageal mucosal damage caused by excessive contact (or exposure) of gastric juice in gastroesophageal lumen. Cerebral ischemia: A syndrome in which the blood supply of the brain is insufficient to meet the metabolic needs of the brain, resulting in a series of symptoms. Ischemic heart disease (IHD): The disease is caused by plaque building up along the inner walls of the arteries of the heart, which narrows the lumen of arteries and reduces blood flow to the heart.

#### 2.2.4 Statistical analysis

The qualitative indexes in logistic regression model were age, AIG, gastroesophageal reflux, coronary heart disease, cerebral ischemia, and sex. The quantitative indexes are blood pressure, RBC, and HCY value. According to the abovementioned criteria, the quantitative index is transformed into qualitative index.

A binary logistic regression analysis model is constructed to explore the relationship between AIG and related indicators. We describe the dependent variable as AIG and the independent variable as PCA, age, gastroesophageal reflux, hypertension, coronary heart disease, cerebral ischemia, decreased RBC, increased HCY, and gender.

Statistical analysis was performed using SPSS version 19.0 software (IBM Corp.). The difference was statistically significant with *P* < .01. The counting data of between groups were compared by chi‐square test.

## 3 RESULT

### 3.1 Comparison of PCA‐positive detection rate between the population in the department of gastroenterology and the physical examination population

Among the 6269 patients who were examined for PCA, 2243 were PCA positive. The positive detection rate was 35.80% (2243/6269), of which 1015 were male and 1228 were female, male: female = 1:1.21. PCA‐positive rate was 11.25% (45/400) in 400 healthy people, which was much lower than that in the visiting population, *χ* ^2^ = 100.53, *P* < .01, (Table 1).

**Table 1:** Comparison of the positive rate of parietal cell antibody (PCA) between the patients in the Department of Gastroenterology and the healthy people

	Patients in Department of Gastroenterology	Physical examination group	*χ* ^2^	*P*
Total	6269	400		
PCA‐positive population	2243 (35.80%)	45 (11.25%)	100.53	<.01

### 3.2 Comparative analysis of gastroscopy results between PCA‐positive and PCA‐negative population

Among the 6269 patients who were tested for PCA, 3017 were concurrently undergoing esophagogastroduodenoscopy (EGD), of which 926 were positive for PCA and 2091 were negative. Of 926 PCA‐positive cases, 311 were diagnosed as atrophic gastritis by EGD, accounting for 311/926 = 33.59%; Of 2091 PCA‐positive cases, 190 were diagnosed as atrophic gastritis by EGD, accounting for 190/2091 = 9.09%. There was a significant difference between them, *χ* ^2^ = 278.15, *P* < .01. The accurate diagnosis rate of AIG in PCA‐positive population was much higher than that in PCA‐negative population. Among the PCA‐positive people, 615 cases were not diagnosed as AIG by EGD, accounting for 615/926 = 66.41%, and there were potential factors for the development of AIG in this population (Table 2).

**Table 2:** Comparative analysis of gastroscopy results between parietal cell antibody (PCA)‐positive and PCA‐negative population

	Total	PCA positive	PCA negative	*χ* ^2^	*P*
Population tested for PCA	6269	2243 (35.80%)	4026 (64.22%)		
PCA detection + Gastroscopy	3017	926 (30.69%)	2091 (69.30%)		
Gastroscope diagnosis of AIG population	501	311 (62.07%)	190 (37.92%)	58.45	<.01
AIG diagnosis rate	16.61%	33.59%	9.09%	278.13	<.01
PCA‐positive people and not diagnosed as AIG		66.41%			

### 3.3 Investigation and analysis of clinical symptoms and medical history in PCA‐positive population

A retrospective analysis of clinical data of 2243 PCA‐positive people revealed that the incidence of gastroesophageal reflux was higher, accounting for 1468/2243 = 65.45%; medical history of cerebrovascular diseases such as hypertension, IHD, and cerebral ischemia accounted for a higher proportion, 1831/2243 = 81.63%, 346/2243 = 15.43%, and 709/2243 = 31.61%, respectively, which was significantly higher than that of negative and physical examination control groups. The proportion of gastroesophageal reflux, hypertension, IHD, and cerebral ischemia in the negative group was significantly higher than that in the physical examination group (Table 3).

**Table 3:** Investigation of medical history of parietal cell antibody (PCA)‐positive population

Medical history	Positive group (n = 2243)	Negative group (n = 400)	Physical examination group(n = 400)	$\chi_{1}^{2}$	$\chi_{2}^{2}$	$\chi_{2}^{3}$	*P*_1_	*P*_2_	*P*_3_
Gastroesophageal reflux	1468 (65.45%)	63 (15.75%)	29 (7.25%)	344.02	468.16	14.20	<.01	<.01	<.01
Hypertension	1831 (81.63%)	169 (42.25%)	121 (30.25%)	285.98	464.14	12.46	<.01	<.01	<.01
Ischemic heart disease	346 (15.43%)	31 (7.75%)	4 (1.00%)	16.35	61.49	21.76	<.01	<.01	<.01
Cerebral ischemia	709 (31.61%)	26 (6.5%)	10 (2.5%)	106.61	142.25	7.45	<.01	<.01	<.01

$\chi_{1}^{2}$, *P*_1_:Comparison between PCA‐positive groups and PCA‐negative groups;

$\chi_{2}^{2}$, *P*_2_:Comparison between PCA‐positive groups and physical examination groups;

$\chi_{2}^{3}$, *P*_3_:Comparison between PCA‐negative groups and physical examination groups.

### 3.4 Investigation on related Indexes of laboratory test in PCA‐positive population

According to the investigation and analysis of blood routine test, liver function, and kidney function test items in 2243 PCA‐positive people, it was found that the decrease in RBC and the increase in HCY were 792/2243 = 35.31% and 1551/2243 = 69.15%, respectively, which were significantly higher than those in the negative control and physical examination group (Table 4).

**Table 4:** Comparative analysis of laboratory test indicators in parietal cell antibody (PCA)‐positive population

Detection index	Positive group(n = 2243)	Negative group (n = 400)	Physical examination group(n = 400)	$\chi_{1}^{2}$	$\chi_{2}^{2}$	$\chi_{2}^{3}$	*P*_1_	*P*_2_	*P*_3_
Decreased RBC	792 (35.31%)	57 (14.25%)	9 (2.25%)	69.05	175.66	38.05	<.01	<.01	<.01
Increased HCY	1551 (69.15%)	123 (30.75%)	73 (18.25%)	215.54	371.22	16.89	<.01	<.01	<.01

$\chi_{1}^{2}$, *P*_1_: Comparison between PCA‐positive groups and PCA‐negative groups;

$\chi_{2}^{2}$, *P*_2_: Comparison between PCA‐positive groups and physical examination groups;

$\chi_{2}^{3}$, *P*_3_: Comparison between PCA‐negative groups and physical examination groups.

### 3.5 Logistic regression assesses correlation between AIG and PCA

Binary Logistic regression was used to assess the relationship between AIG and PCA, age, AIG, gastroesophageal reflux, hypertension, IHD, cerebral ischemia, decreased RBC, increased HCY, and gender. Finally, the logistic regression model is statistically significant, *χ* ^2^ = 211.492, *P* < .001. The model can correctly classify 95.3% of the research objects. The sensitivity of the model is 59.1% and the specificity is 95.3%. The analysis in the model showed that if PCA was positive, the risk of AIG increased by 18.521 times. These results suggest that PCA is an independent risk factor for AIG (Table 5).

**Table 5:** Logistic regression assesses correlation between AIG and parietal cell antibody (PCA)

Omnibus test of model coefficients			
	*χ* ^2^	*df*	Sig.
Step 1			
Step	211.492	9	.000
Block	211.492	9	.000
Model	211.492	9	.000

Classification table^a^					
	Actual measurement		Prediction		
			AIG		Correct percentage
			0	1	
Step 1	AIG	0	405	20	95.3
		1	47	68	59.1
	Overall percentage				87.6

Variables in the equation									
								95% confidence interval of EXP (B)	
		B	S.E	Wals	*df*	Sig.	Exp(B)	Lower limit	Upper limit
Step 1^b^	Age	−0.007	.011	0.408	1	.523	0.993	0.971	1.015
	Gastroesophageal reflux	−0.351	.345	1.039	1	.308	0.704	0.358	1.383
	Hypertension	−2.605	.383	46.266	1	.000	0.074	0.035	0.157
	Ischemia heart disease	−0.108	.418	0.066	1	.797	0.898	0.396	2.036
	Cerebral ischemia	−0.351	.350	1.003	1	.317	0.704	0.355	1.399
	Decreased RBC	2.293	.303	57.332	1	.000	9.907	5.472	17.938
	Increased HCY	0.032	.305	0.011	1	.915	1.033	0.568	1.878
	Gender(1)	0.442	.280	2.496	1	.114	1.556	0.899	2.693
	PCA	2.919	.378	59.537	1	.000	18.521	8.824	38.875
	Constant	−2.111	.706	8.945	1	.003	0.121		

a The cutoff value is .500.

b The variables entered in step 1 as follows: PCA, Age, Gastroesophageal reflux, Hypertension, Ischemia heart disease, Cerebral ischemia, Decreased RBC, Increased HCY, and Gender.

## 4 DISCUSSION

The incidence of chronic gastritis in China is as high as 60%.^3^ Chronic gastritis is divided into non‐atrophic gastritis and atrophic gastritis according to morphological changes. However, due to the low attention of patients and the limitations of EGD and biopsy, the sensitivity of microscopic diagnosis of atrophic gastritis^4^ was reduced, and the diagnosis was greatly delayed. How to make early diagnosis is a difficult problem in this field. Chronic atrophic gastritis can be divided into type A and B according to different etiologies. Type A atrophic gastritis—AAG, is the main type of atrophic gastritis. It is marked by the presence of autoantibodies such as PCA and anti‐internal factor antibodies in serum. Early detection is very important for the early discovery of AIG. PCA is the initiator of type A atrophic gastritis. Studies have shown that PCA exists in the serum of patients earlier than the typical symptoms of the disease.^5^ Literature studies have shown that PCA is an early marker of gastric mucosal atrophy.^6^ In our study, we found that the detection rate of PCA in the department of gastroenterology was as high as 35.80%. The proportion of positive people diagnosed with atrophic gastritis by microscopy was much higher than that of negative group. Most of the PCA‐positive population failed to diagnose AIG under gastroscopy. However, with the progress of the disease, it may develop into AIG. It is necessary to conduct in‐depth research to further reveal the predictive value of PCA for disease. The clinical guidance of this survey results is clear.

In our study, the standard for admission was the occurrence of one or more digestive system symptoms in the population, so the current study did not analyze the common digestive system symptoms such as nausea, vomiting, anorexia, acid reflux, belching, abdominal pain, and abdominal distention. However, the incidence of gastroesophageal reflux is as high as 65.45%, which should cause high vigilance. This may be related to the abnormal peristalsis caused by hardening, inflammation, ulcer, and even atrophy of gastric body caused by PCA.

In our study, we found that the proportion of increased HCY in PCA‐positive population was as high as 69.15%, and the proportion of cardio‐cerebral vascular disease such as hypertension was also higher. The main mechanism of this phenomenon is that PCA damages gastric parietal cells, resulting in the decrease of hydrochloric acid endocrine factors, vitamin B12 absorption barrier, methionine circulation obstruction, so that HCY cannot be converted into methionine and excreted out of the body, which accumulates in the blood stream and causes hyper HCY.^7^ High levels of HCY in plasma can damage vascular endothelial cells, accelerate atherosclerosis, increase vascular resistance, and lead to H‐type hypertension. Studies on the mechanism of thrombosis have found that HCY damages the vascular endothelium, promotes the proliferation of smooth muscle cells, and enhances platelet function, leading to vascular sclerosis, narrowing of the lumen, slowing down of blood flow, and promoting thrombosis.^8^ Hypertension is a basic disease of cardiovascular and cerebrovascular diseases. H‐type hypertension accounts for up to 75% of hypertensive patients,^9^ which seriously endangers people's health. The results of this survey should be highly valued.

Gastrointestinal diseases can induce anemia through different mechanisms.^10^ Pernicious anemia caused by PCA‐related atrophic gastritis is closely related to the following aspects: Firstly, the decrease of gastric parietal cells leads to the decrease of gastric acid secretion. Fe^3+^ in food cannot be reduced to Fe2^+^, which can be absorbed by the body. It affects the synthesis of hemoglobin and leads to iron deficiency anemia; secondly, the decrease of gastric parietal cells leads to the decrease of endocrine factors secretion and absorption disorder of vitamin B_12_, which leads to the decrease of tetrahydrofolate synthesis, the disorder of nucleic acid synthesis, the obstruction of cell division, and finally formation of megaloblastic anemia. As the disease progresses, a large number of glands begin to atrophy, and a variety of nutrients are mal absorbed. Erythropoiesis is further affected, and a vicious cycle is formed.^11^ Oral drug treatment for pernicious anemia is not effective, so it needs to be treated by intramuscular injection of vitamin B_12_ for life.^12^ It is reported that iron deficiency anemia occurs earlier than pernicious anemia, and vitamin B_12_ has been deficient for many years before the occurrence of pernicious anemia.^13^ There are also reports of regular intramuscular injection of vitamin B_12_ to reduce the titer of PCA and alleviate related diseases.^14^ It is confirmed that the level of vitamin B_12_ is strongly correlated with the titer of PCA. In this survey, the percentage of decreased RBC in PCA‐positive population is much higher than that in control group, which deserves great attention.

We should attach great importance to gastropathy, and the role of stomach as the first pass of human metabolism is very important. In this study, many kinds of chronic diseases caused by injury of digestive function that PCA induced were discussed and analyzed from the aspects of theoretical basis and clinical practice. We found that PCA is closely related to gastritis, especially type A atrophic gastritis, gastroesophageal reflux, and other digestive system diseases, as well as H‐type hypertension, iron deficiency anemia, megaloblastic anemia, pernicious anemia, and other chronic diseases. The relationship between AIG and PCA was discussed by logistic regression analysis, and it was found that PCA was an independent risk factor for AIG. PCA appears in the serum of patients earlier than clinical symptoms, and early detection is of great value for early warning of a variety of chronic diseases. The detection of APC has little trauma and is widely used in clinic.

The limitation of this study is that there is no longitudinal follow‐up survey of PCA‐positive people. In this study, we found that most of the PCA‐positive people could not be diagnosed as AIG by gastroscopy. This part of the population may develop into AIG as the disease progresses, which belongs to the potential population and needs further study.
